# Nonprofessional Phagocytosis Can Facilitate Herpesvirus Entry into Ocular Cells

**DOI:** 10.1155/2012/651691

**Published:** 2012-03-15

**Authors:** Vaibhav Tiwari, Deepak Shukla

**Affiliations:** ^1^Department of Microbiology and Immunology, Midwestern University, Downers Grove, IL 60515, USA; ^2^Department of Microbiology and Immunology, University of Illinois at Chicago, 1855 W. Taylor Street, LIERI Building, Chicago, IL 60612, USA; ^3^Department of Ophthalmology and Visual Sciences, University of Illinois at Chicago, 1855 W. Taylor Street, LIERI Building, Chicago, IL 60612, USA

## Abstract

Phagocytosis is a major mechanism by which the mediators of innate immunity thwart microbial infections. Here we demonstrate that human herpesviruses may have evolved a common mechanism to exploit a phagocytosis-like entrapment to gain entry into ocular cells. While herpes simplex virus-1 (HSV-1) causes corneal keratitis, cytomegalovirus (CMV) is associated with retinitis in immunocompromised individuals. A third herpesvirus, human herpesvirus-8 (HHV-8), is crucial for the pathogenesis of Kaposi's sarcoma, a common AIDS-related tumor of eyelid and conjunctiva. Using laser scanning confocal microscopy, we show that successful infection of ocular cell types by all the three viruses, belonging to three divergent subfamilies of herpesviruses, is facilitated by induction of F-actin rich membrane protrusions. Inhibitors of F-actin polymerization and membrane protrusion formation, cytochalasin D and latrunculin B, were able to block infection by all three viruses. Similar inhibition was seen by blocking phosphoinositide 3 kinase signaling, which is required for microbial phagocytosis. Transmission electron microscopy data using human corneal fibroblasts for HSV-1, human retinal pigment epithelial cells for CMV, and human conjunctival epithelial cells for HHV-8 are consistent with the possibility that pseudopod-like membrane protrusions facilitate virus uptake by the ocular cells. Our findings suggest a novel mechanism by which the nonprofessional mediators of phagocytosis can be infected by human herpesviruses.

## 1. Introduction

Phagocytosis is essentially a form of endocytosis wherein particles are trapped and enclosed by cell membrane protrusions. Our knowledge of phagocytosis comes mainly from professional phagocytes such as macrophages and neutrophils, which fight against microbial invasion and removal of dead cells [1]. However, in many cases, nonprofessional phagocytes including epithelial cells and fibroblasts of ocular origin have also been shown to possess the ability to phagocytose their adjacent apoptotic cells or spent cell debris [1–3]. Well-known examples include Sertoli cells in testis [3] and the retinal pigment epithelial (RPE) cells in the retina [3]. Recently we demonstrated that herpes simplex virus-1 (HSV-1) has the ability to exploit phagocytosis to promote its entry into corneal fibroblasts [4]. Similar findings have been made with amoebal mimivirus [5, 6]. Nonprofessional phagocytosis is also triggered by the recognition of ligands by corresponding receptors on phagocytosing cells. This results in surrounding of the target particles with a specialized pseudopod-like extension of the plasma membrane. The local reorganization of F-actin underneath the extension and the contractile motors supporting the reorganization provide the driving forces for trapping the particles [2, 7, 8]. Similar to professional phagocytosis by macrophages and neutrophils, nonprofessional phagocytosis also requires phosphoinositide 3 kinase (PI3K) signaling [6].

Herpesviruses are highly prevalent among humans [9]. A vast majority of adult human population is seropositive for multiple herpesviruses, which cause life-long infections and virtually all are capable of causing ocular manifestations [9, 10]. The family of herpesviruses, which may have more than a hundred known members, has been divided into three subfamilies. Among human herpesviruses, alphaherpesvirus subfamily is exemplified by herpes simplex virus-1 (HSV-1), betaherpesvirus subfamily by cytomegalovirus (CMV), and gammaherpesvirus subfamily by human herpesvirus-8 (HHV-8) [9]. The most common eye infections are caused by HSV-1, which is a well-studied cause for herpes stromal keratitis (HSK), a blinding eye disease. In addition HSK is also associated with blepharitis, dendritic keratitis, disciform stromal edema, and conjunctivitis [11]. The involvement of CMV and HHV-8 in ocular diseases is mostly limited to immunocompromised human population which includes AIDS patients and organ transplant recipients [10]. CMV used to cause retinitis in a significant number (30% or more) of AIDS patients. Lately, this situation has been brought under control by introduction of highly active antiretroviral therapy (HAART). However, the ocular problems associated with HHV-8 remain very common among the AIDS patients who often suffer from the tumors of eyelid and conjunctiva [12].

The mechanisms by which herpesviruses enter into host cells vary with individual viruses [4, 13–15]. For instance, all the three herpesviruses discussed previously use separate entry receptors, prefer certain cell types over others for infection and the establishment of latency, and use different mode(s) of entry [16]. In the case of HSV-1, endocytosis and nonprofessional phagocytosis play a dominant role in infection of many cell types [4, 14, 17]. Recent studies have indicated that HSV-1 entry may be atypical endocytosis since it is not mediated by formation of clathrin-coated pits or caveolae and it may or may not be pH-dependent [18, 19]. CMV and HHV-8, in contrast, may enter into cells by clathrin-coated endocytic cup formation and the entry is pH dependent [15]. While the significance of endocytosis may be known, it is not clear how herpesviruses infect cells of ocular origin, many of which are immune privileged [20]. It is also unknown if actin cytoskeleton plays a direct role in the initiation of infection, and likewise, the significance of pseudopod-like protrusions in entry process has not been described for herpesviruses. Here we demonstrate a unique commonality in terms of entry mechanism among three representative members of herpesvirus subfamilies. We show that the entry of the viruses into target cell types of ocular origin is facilitated by F-actin containing pseudopod-like membrane protrusions. Similar to phagocytosis, this entry mechanism is also controlled by PI3K signaling. The protrusions formed during nonprofessional phagocytosis may facilitate viral uptake since inhibition of protrusion formation significantly inhibits viral entry or uptake into cells. Our findings also demonstrate a possible way by which herpesviruses may have evolved to escape from neutralization by the innate mediators of phagocytosis.

## 2. Materials and Methods

### 2.1. Cell Cultures

 Retinal pigmental epithelial (RPE) cells were grown in Dulbecco's modified Eagle's (DMEM) medium supplemented with 10% fetal bovine serum (FBS) containing penicillin and streptomycin [21]. Primary cultures of human corneal fibroblasts (CFs) cells were grown in DMEM medium containing 10% FBS and 5% calf serum (CS) as previously described [22]. Primary cultures of human conjunctival epithelial (HCE) cells were kindly provided by Dr. Ilene K. Gipson (Schepens Eye Research Institute; Harvard Medical School). HCE cells were cultured on GIBCO keratinocytes serum-free medium with supplied BPE (bovine pituitary extract) and EGF (0.2 ng/mL) [23]. CMV virus was detected by a monoclonal antibody specific for glycoprotein B (Virostat Inc., Portland, ME). HHV-8 virus was detected by a monoclonal antibody specific for HHV-8 envelope glycoprotein K8.1A and FITC-conjugated secondary antibody.

### 2.2. Viruses

The *β*-galactosidase-expressing recombinant HSV-1 gL86, GFP-expressing HSV-1 (K26GFP-HSV-1), cytomegalovirus (CMV; Towne strain RC256), and HHV-8 virus (wild-type and rKSHV.152) were used in this study. HSV-1 gL86 was propagated in gL complementing cell line (79B4 cells). GFP-expressing HSV-1 (K26GFP) virus was grown in Vero cells [4]. The viruses were purified by sucrose density gradient as previously described [22].

### 2.3. Electron Microscopy

CF, RPE, and HCE cells cultured in Lab-Tek chamber slides and in Anopore wells (Nalge Nunc) (approximately 1.5 × 10^5^ cells/well) were infected with purified HSV-1, CMV, and HHV-8 at 50–100 MOI for 90–120 min as previously described [4]. Infected cells were rinsed with serum-free buffer and fixed with 1% osmium tetroxide for 10 s, immediately followed by the aldehyde fixation for 1 hr (2.5% glutaraldehyde, 2% paraformaldehyde in 100 mM cacodylate buffer, pH 7.4). Cells were rinsed three times for 5 min with 100 mM cacodylate buffer, postfixed for 1 hr in 1% osmium tetroxide, stained with uranyl acetate, dehydrated through a graded ethanol series, and finally embedded using Embed. For transmission electron microscopy (TEM) (JEM-1220; JEOL USA Inc.) images were captured at 1,000–600,000x, point-0.36 nm (3.6 A), and lattice-0.2 nm (2 A) at room temperature, ACC voltage 40–120 kV using a Gatan camera (Digital CCD; Gatan Inc.), and Gatan Digital Micrograph (DM) v2.5 acquisition software.

### 2.4. Indirect Immunofluorescence

Target CF, RPE, and HCE cells cultured in Lab-Tek chamber slide were infected with herpes viruses for 90–120 minutes. The cells were then stained. For cell surface immunofluorescence, the cells were incubated with primary antibodies at 4°C for 45 min, washed 10 times with cold PBS, and fixed with acetone for 10 min at −20°C. HHV-8 and CMV viruses were detected by anti-gB (CMV) and anti-K8.1A (HHV-8) antibodies and FITC-conjugated secondary antibodies. Actin fibers were also stained with 10 nM rhodamine-conjugated phalloidin (Molecular Probes, Carlsbad, California, USA) to compare the number of actin-rich protrusion produced in the infected cells compared to uninfected cell. The cells were washed before mounting in Vectashield mounting medium (Vector Laboratories, Inc. Burlingame, CA). Leica confocal microscope SP2 was used for imaging.

### 2.5. Viral Entry Assay

Viral entry assay was based on quantitation of *β*-galactosidase expressed from HSV-1 genome by soluble O-nitro-phenyl-*β*-D-galactopyranoside (ONPG, Pierce Biotechnology), 3 mg/mL. Similarly CMV *β*-galactosidase-expressed reporter virus from ATCC was used. HHV-8 virus entry was determined by fluorescence readout assay. CF, RPE, and HCE were either mock treated or treated with medium containing Cyto D (0.5 and 1.0 *μ*g/mL), Lat B (0.25–2.5 *μ*M), PI3-kinase inhibitor (LY294002), and in active compound LY303511. In parallel experiment CF, RPE, and HCE cells were transfected with PI3K dominant-negative expression plasmid (ΔiSH2) or with control plasmid followed by the viral infection [24].

## 3. Results and Discussion

**Figure 1:**
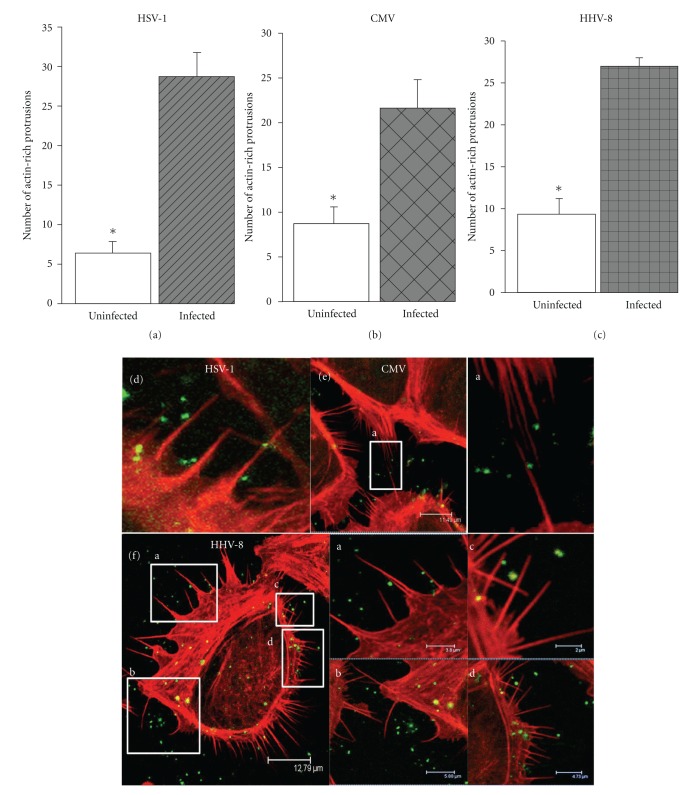
Herpesvirus infection leads to increase in the number of F-actin-rich projections. (a)–(c) Counting of the projections on per cell basis was done 15–30 min before and after the addition of the virus indicated (Herpes simplex virus type-1; (HSV-1), Cytomegalovirus; (CMV) and Human herpesvirus-8; (HHV-8)). In this and all other subsequent experiments cells used included corneal fibroblasts (CFs) (a) for HSV-1, human retinal pigment epithelial (RPE) cells for CMV (b), and primary cultures of human conjunctival epithelial (HCjE) for HHV-8 (c) infection. Four independent experiments were conducted to record the data. Numbers of actin-rich protrusions are presented as means with error bars showing standard deviation. (d)–(f) Confocal images of cells challenged with indicated viruses are shown. Virally infected cells were fixed, permeabilized, and stained for F-actin with 10 nM rhodamine-conjugated phalloidin before mounting (Vectashield mounting medium). All confocal (Leica SP2) images were captured at 63x objective. Magnified views of the images in the boxes are shown next to them and labeled identically. CMV and HHV-8 viruses were identified by monoclonal antibodies against CMV envelope glycoprotein gB and HHV-8 K8.1A (1 : 50) followed by secondary antibody treatment conjugated to FITC.

We began our investigation by monitoring morphological changes that occur in ocular cells upon infection with herpesviruses. The original goal was to highlight differences in the way the herpesviruses, belonging to three distinct subfamilies, infect their target cells of ocular origin. We used primary human corneal fibroblasts (CFs), human conjunctival epithelial (HCE), and human retinal pigment epithelial (RPE) cells for infection with green fluorescent protein expressing HSV-1 (K26GFP), CMV (AD169), and HHV-8 (courtesy of J. Vieira, University of Washington, Seattle, WA), respectively. To our surprise, infection of three different cell-types by three different herpesviruses resulted in a common morphological change, which was represented by a clear enhancement in the number of F-actin rich plasma membrane protrusions (Figures 1(a)–1(c)). The protrusions were 1.0–10 *μ*m large and often uniformly distributed along the surface of individual cells (Figures 1(d)–1(f)). Protrusions were observed during HSV-1 invasion of CF (Figures 1(a) and 1(d)), CMV invasion of RPE cells (Figures 1(b) and 1(e)), and HHV-8 invasion of HCE cells (Figures 1(c) and 1(f)).

**Figure 2:**
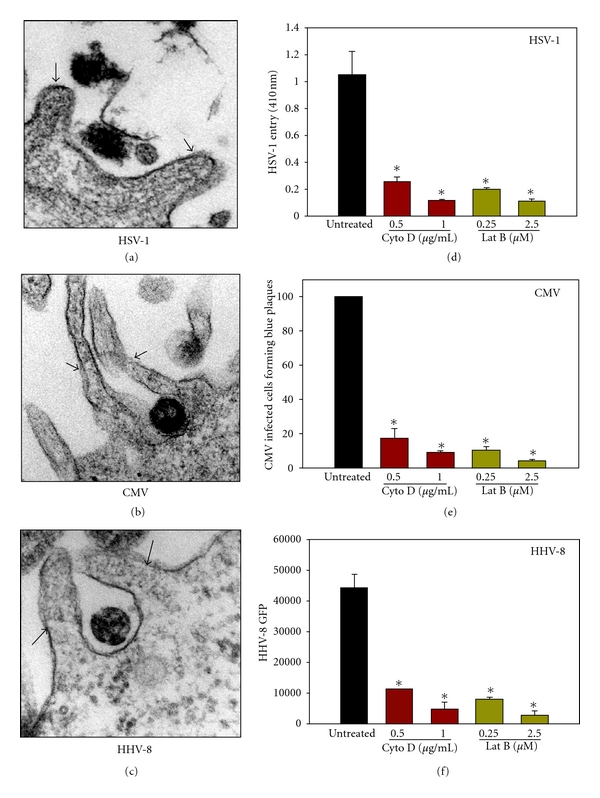
Herpesviruses can be internalized by membrane protrusions and blocking of protrusion formation blocks herpesvirus entry. (a)–(c) High-resolution transmission electron micrographs (TEMs) of human ocular cells infected with herpesviruses are shown. Arrowheads in the panels indicate the association of viruses to cellular projections. (d)–(f) F-actin-dependent uptake of herpesviruses into ocular cells. Effects of actin depolymerizers (cytochalasin D; Cyto D and latrunculin b; Lat B) on virus entry into ocular cells (CF (d) for HSV-1, RPE cells for CMV (e), and HCjE for HHV-8 (f)) were examined. Cells were treated with Cyto D or Lat B at concentrations indicated for 90 min before exposure to the virus indicated. Asterisks indicate significant differences from controls (*P* < 0.05, *t*-test *n* = 14, error bars represent SD).

 Since a phagocytosis-like uptake for HSV-1 has been suggested and many ocular cell-types can perform phagocytosis [4], we decided to generate ultrastructural evidence to support the possibility that projections may help with virus entrapment as well. Thus, as described previously [4], transmission electron microscopy (TEM) was performed with virally infected ocular cells (CF for HSV-1, RPE for CMV, and HCE for HHV-8). Again, a possible significance of protrusions that were formed by extension of the plasma membrane (continuity of the plasma membrane was visible in all cases) was indicated by the presence of virus particles next to them (Figure 2). HSV-1 (Figure 2(a)), CMV (Figure 2(b)), and HHV-8 (Figure 2(c)) were all seen present inside the cups formed by plasma membrane protrusions. In many cases, protrusions seemed to entrap single virus particles (Figures 2(b) and 2(c)).

 To generate biochemical evidence in support of the role of membrane protrusions in productive viral uptake, inhibitors of F-actin polymerization were examined for their ability to block the infection. The inhibitors used were cytochalasin D (Cyto D) and latrunculin B (Lat B), both block F-actin polymerization that is required for protrusion formation by the plasma membrane [25, 26]. For HSV-1, virus uptake was determined by colorimetric analysis of entry of a *β*-galactosidase expressing reporter virus HSV-1(KOS) gL86. As previously described, transcription of early virus genes allows expression of *β*-galactosidase activity that is read by a spectrophotometer after addition of its soluble substrate ONPG [27]. A similar *β*-galactosidase expression-based plaque assay was used for CMV and GFP expression in infected cells was used as a marker for virus infection of HCE cells by HHV-8 [4]. It was seen in all cases that at previously determined dosages, both inhibitors have significant negative effects on viral infection. About 50–80% HSV-1 entry into CF was blocked by the inhibitors (Figure 2(d)). Similarly, 40–75% of CMV-infected cells treated with the drugs significantly inhibited viral entry (Figure 2(e)) and 40 to 60% reduction in GFP expressing cells was observed in Cyto-D and Lat-B-treated HCE cells (Figure 2(f)). It is therefore likely that membrane protrusion formation is a common and important requirement for herpesvirus infection of the ocular cells.

**Figure 3:**
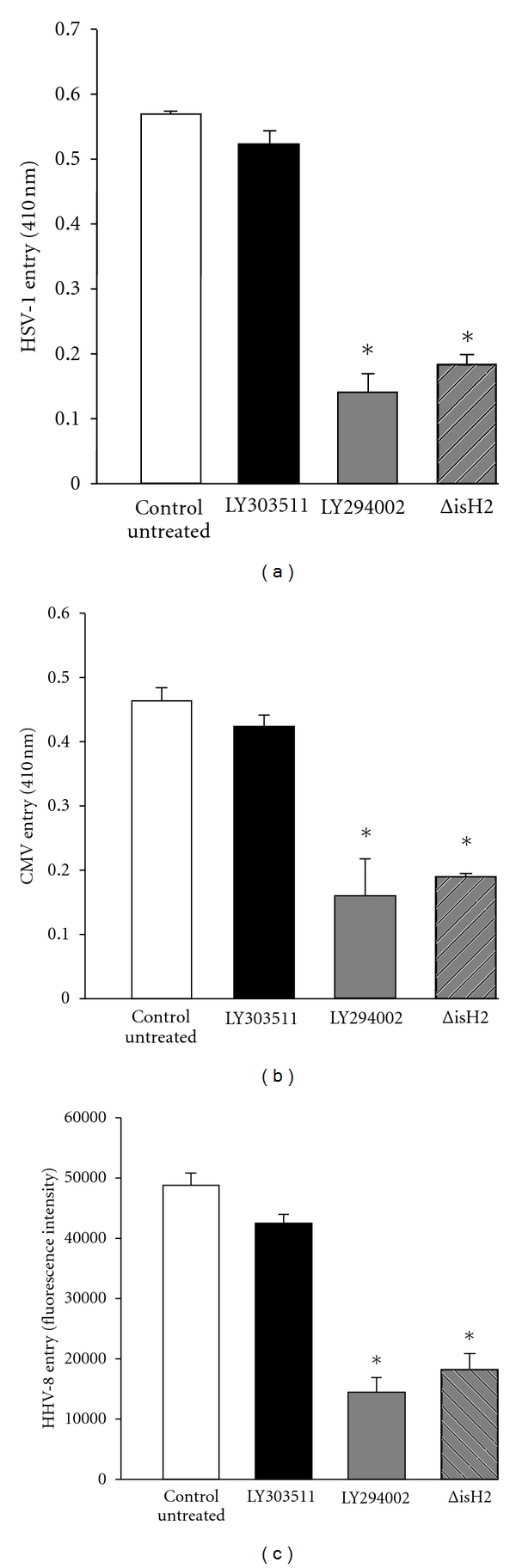
(a)–(c) Inhibition of PI3 Kinase signaling pathway negatively affects herpesvirus entry into cell. Cells were either treated with the compound indicated or transfected with PI3-dominant negative plasmid (ΔiSH2) followed by infection with HSV-1 (a), CMV (b), and HHV-8 (c). Results from viral entry assay are shown.

 Finally, to add more evidence in favor of the involvement of membrane protrusion formation and phagocytosis in herpesvirus uptake by ocular cells, we decided to block PI3K-mediated signaling, which is required for phagocytic cup formation [6]. As shown in Figures 3(a)–3(c), ocular cells pretreated with a PI3Kinase inhibitor (LY294002) [28, 29] showed decreased entry by HSV-1 (Figure 3(a)), CMV (Figure 3(b)), and HHV-8 (Figure 3(c)). A similar level of decrease was also seen when the cells were first transfected with an expression construct for a dominant-negative PI3K mutant lacking the p110-catalytic subunit-binding domain (ΔiSH2) of PI3K [30] and then infected with the herpesviruses (Figures 3(a)–3(c)). In all cases it was clear that PI3K signaling was required for efficient infection by the viruses. To determine the specificity of PI3K blocking, we used a compound LY303511 (Calbiochem Inc.) that is highly related to LY294002. It was again clear that the former had no significant effect on HSV-1 (Figure 3(a)), CMV (Figure 3(b)), or HHV-8 (Figure 3(c)) entry.

In summary, we demonstrate some unique features of herpesvirus infection of the cells of ocular origin. It is evident that the ocular cells may respond to the three representative herpesvirus subfamily members by inducing F-actin-based membrane protrusions. Any blockage of protrusion formation greatly restricts viral entry (Figure 1) and inhibition of PI3K signaling, a requirement for F-actin remodeling during phagocytosis, also blocks herpesvirus entry into the ocular cells (Figure 3). PI3Ks are cellular heterodimeric enzymes that consist of a regulatory subunit (p85) activated by tyrosine phosphorylation, which recruits inositol phospholipids that are phosphorylated by the catalytic subunit (p110) [31–34]. The lipids then serve as second messengers that control phosphorylation of other kinases such as Akt/PKB, some protein kinase C isoforms, cyclic AMP-dependent protein kinase A, and the ribosomal S6 kinases p70 and p85 [35]. The ability of PI3K to regulate pathways important for phagocytosis coupled with the ability of herpesviruses to modulate its activity could be an important mechanism by which the viruses exploit phagocytosis for entry. Our observation is also supported by the fact that additional intracellular signaling that may be needed for protrusion formation, such as activation of Rho family of GTPases, has already been reported with many herpesviruses [15, 36]. The most significant contribution of our study is to demonstrate the possibility that involvement of membrane protrusions may be common, and perhaps crucial, for herpesvirus infection of the ocular cell types. Herpesviruses tend to spread by asymptomatic shedding, which allows normal looking individuals to transmit viruses to healthy individuals [37]. In the absence of effective vaccines against many herpesviruses, new and effective wide spectrum prophylactics are desperately needed to prevent an alarming rate of increase in herpesvirus transmission. In this regard, our study identifies an important common target for future intervention against multiple herpesviruses. It may be that not just herpes but many additional unrelated viruses also begin their journey to infection by interacting with membrane protrusions and exploiting phagocytosis or a very similar mechanism for infection. Our ultimate goal is to identify how the virions successfully exit from degradation in phagolysosomes.
